# MiRNA-Target Interaction Reveals Cell-Specific Post-Transcriptional Regulation in Mammalian Cell Lines

**DOI:** 10.3390/ijms17010072

**Published:** 2016-01-08

**Authors:** Varun Kulkarni, Afsar Raza Naqvi, Juhi Raju Uttamani, Salvador Nares

**Affiliations:** Department of Periodontics, College of Dentistry, University of Illinois at Chicago, 458 Dent MC 859, 801 S. Paulina, Chicago, IL 60612, USA; vrun_k@hotmail.com (V.K.); afsarraz@uic.edu (A.R.N.); juttam2@uic.edu (J.R.U.)

**Keywords:** microRNA, gene regulation, cell lines, Ago-2 (Argonaute-2)

## Abstract

MicroRNAs are 18–22 nucleotides long, non-coding RNAs that bind transcripts with complementary sequences leading to either mRNA degradation or translational suppression. However, the inherent differences in preferred mode of miRNA regulation among cells of different origin have not been examined. In our previous transcriptome profiling studies, we observed that post-transcriptional regulation can differ substantially depending on the cell in context. Here we examined mechanistic differences in the regulation of a let-7a targeted (wild type) or resistant (mutant) engineered renilla transcript across various mammalian cell lines of diverse origin. Dual luciferase assays show that compared to mutant (mut), the reporter gene containing wild type (wt) let-7a binding sites was efficiently suppressed upon transfection in various cell lines. Importantly, the strength of miRNA regulation varied across the cell lines. Total RNA analysis demonstrates that wt renilla mRNA was expressed to similar or higher levels compared to mut suggesting that translation repression is a predominant mode of miRNA regulation. Nonetheless, transcript degradation was observed in some cell lines. Ago-2 immunoprecipitation show that miRNA repressed renilla mRNA are associated with functional mi-RISC (miRNA-RNA induced silencing complex). Given the immense potential of miRNA as a therapeutic option, these findings highlight the necessity to thoroughly examine the mode of mRNA regulation in order to achieve the beneficial effects in targeting cells.

## 1. Introduction

The discovery of microRNAs (miRNA) depicts one of the most important advances in our understanding of the mechanisms regulating gene expression. Since the identification of the first miRNAs in *C. elegans* in 1993 [1], over 2500 miRNAs controlling the mammalian genome have been discovered [2]. These miRNAs regulate various biological systems in mammals including developmental and physiological networks [3]. MiRNAs are 18–22 nucleotides (nts) long, non-coding RNAs that post-transcriptionally regulate mRNA stability and control the translation efficiency of their target genes [4,5]. Primarily, all the cellular and molecular functions are controlled by miRNAs and as many as 60% of all human mRNAs are regulated by miRNA [6]. Post-transcriptional regulation of mRNA targets by miRNAs and their deregulation has been linked to a number of disease processes [3,7].

The biogenesis and mode of action of miRNA have been well characterized [8,9]. The native miRNAs called the primary-miRNAs (pri-miRNA) are situated in the nucleus where they are processed by the Drosha and DiGeorge syndrome critical region 8 (DGCR8) complex into precursor-miRNA (pre-miRNA). Pre-miRNA is a double-stranded RNA hairpin structure, around 70 nucleotides in length that is exported to the cytoplasm through Exportin-5 and is further processed by Dicer into a 22 nts long double stranded RNA. One of the strand of duplex RNA (guide strand) directs RNA-induced silencing complex (RISC) to exert repressive function on its target mRNA [8,10]. MiRNAs bind to complementary sequences in the 3′-untranslated region (3′-UTR) of mRNAs to regulate gene expression by inhibiting protein translation and/or causing mRNA degradation [11]. Due to imperfect complementary binding by miRNAs, a single miRNA can potentially bind to >100 different mRNAs [11,12]. In turn, one gene can be simultaneously regulated by multiple miRNAs [13].

The mammalian structural organization consists of a variety of cell types that are characterized by their physiological properties and location. To a great extent, the identities of these cell types are determined by their patterns of gene expression. MiRNAs regulate genes involved in virtually all physiologic processes while dysregulated miRNA expression and function contributes towards the pathogenesis of numerous diseases. Recent mammalian cell line studies have demonstrated that some miRNAs participate in fine-tuning the production of their targets, both at the mRNA as well as the protein level and play an important role as genetic expression regulators [13,14]. Global protein expression studies in human cells have revealed that translation repression is a predominant mode of miRNA regulation followed by the degradation of miRNA bound transcripts [13,14].

In our previous study we reported dramatic transcriptome-wide changes in human primary dendritic cells (DC) challenged with lipopolysaccharide (LPS) while, macrophages (Mφ) under similar challenge exhibited comparatively less significant changes [15]. We also observed that miRNA or control miRNA mimic-transfected DC show remarkably altered mRNA expression of various genes examined while, only few were impacted in Mφ [16]. We hypothesize that cells may differ in their mode of miRNA regulation. Currently there exist gaps in our understanding of how the posttranscriptional mechanism of gene expression works and how deregulated miRNA expression can link to the etiology of numerous diseases including cancer. The aim of this study is to determine if different cells lines expressing a similar miRNA target would pursue similar or alternate modes of miRNA regulation. Our study also explored the potential for maneuvering miRNA levels to modify gene expression in a cell-specific manner.

## 2. Results

### 2.1. High Transfection Efficiency Established in All Cell Lines

**Figure 1:**
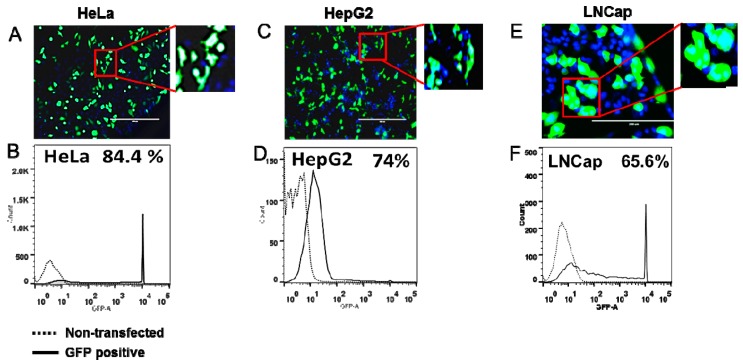
Transfection standardization in cell lines. Transfection efficiency of cells was assessed by transfection with GFP expressing plasmid. Representative images of GFP expressing (**A**) HeLa, (**C**) HepG2 and (**E**) LNCap cells 24 h post-transfection. Flow cytometer analysis showing GFP positive (**B**) HeLa, (**D**) HepG2 and (**F**) LNCap cells. Scale bar, 200 µm.

In this study, we selected 10 cell lines viz., Hep G2, HeLa, HEK-293, COS-7, NIH/3T3, C2C12, U2OS, LNCaP, A549 and HUVEC that have different cellular origins and are derived from human, monkey or mouse. Cells were transfected with pGLOMAX and GFP expression visualized under fluorescence microscopy and quantified by flow cytometry. Figure 1 shows representative images of HeLa and HepG2 cells (A and C) and results from flow cytometric analysis (B and D). Quantitative values of GFP positive cells by flow cytometry demonstrate high transfection efficiency in the range of ~60–90% in all cell lines examined (Table 1).

**Table 1:** Cell lines used in this study and their different origin. Transfection efficiency was determined by transfection with GFP expressing plasmid and analyzed by flow cytometry.

Cell Line	Origin	Transfection Efficiency (%)
HEK293	Human embryonic kidney cell line	90
HeLa	Human cervical cancer cell line	84.4
HepG2	Human liver cancer cell line	74
LNCap	Human prostate cancer cell line	65.8
U2OS	Human osteosarcoma cell line	66
NIH/3T3	Mouse embryonic fibroblast cell line	68.8
A549	Human lung cancer cell line	64.5
C2C12	Mouse myoblast cell line	62.2
Cos-7	Monkey fibroblast-like cell line	60.2
Huvec	Human umbilical vein endothelial cells	60.8

### 2.2. Strength of Translational Suppression Varies with Cell Type

**Figure 2:**
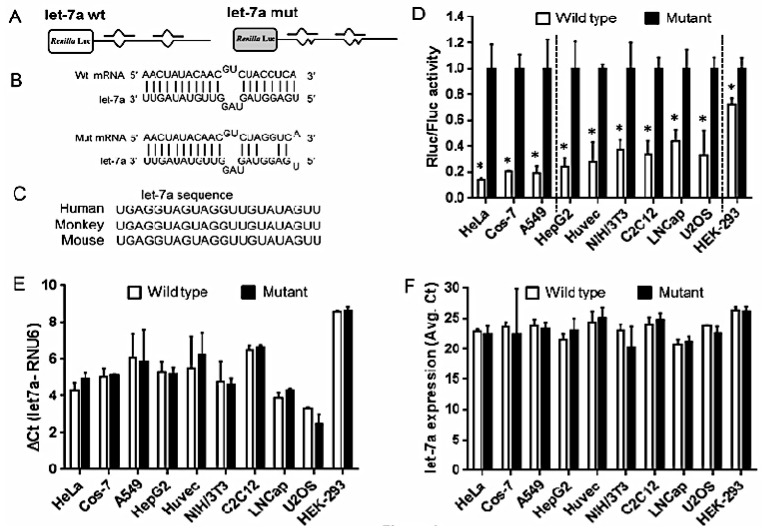
Let-7a mediated suppression of renilla activity varies across cell lines. Schematic drawing showing pRLTK-let-7a constructs with two tandem (**A**) wild-type (wt) and (**B**) mutant (mut) let-7a binding sites downstream to renilla luciferase; (**C**) Cells were co-transfected with pRLTK-let 7a wt or mut and pGL3 constructs; (**D**) Firefly and renilla luciferase activities were measured consecutively using the dual luciferase assays 36 h after transfection. Ratios of renilla and firefly values are presented as mean ± SD from three independent experiments performed in quadruplicates. Value of let-7a mutant construct was set at 1. Dashed lines separate cell lines based on percentage of translation repression: 80% or more (**left**), 25%–60% (**middle**) and less than 25% (**right**). Let-7a expression in not impacted in cells co-transfected with pRLTK-let 7a wt or mut and pGL3 constructs; (**E**) Real-time RT-PCR show the relative expression of let-7a in cells. RNU6B was measured in parallel and used to normalize the expression level of let-7a in each experiment. Values represent means and error bars represent the SD; (**F**) *C*_t_ values of let-7a expression shows no significant difference. Values are presented as mean ± SD from three independent experiments. SD value of some samples is too small and therefore not visible on histograms. * *p* value < 0.05, Students *t*-test.

To evaluate the mode of miRNA regulation, we used pRLTK plasmid that expresses the renilla reporter gene. The renilla gene is engineered with two tandem let7a wild type or mutant binding sites (Figure 2A,B) [17]. Let-7a is highly conserved miRNA and the sequence is 100% identical in humans, monkey and mouse (Figure 2C). This miRNA is ubiquitously expressed at high levels across various cell types. Cells were co-transfected with wt or mut and firefly expressing pGL3 control vector. In all the cell lines tested, dual luciferase results show significant reduction in wild type renilla activity compared to mutant (Figure 2D). This indicates functional interaction of let-7a and its target renilla mRNA. Interestingly, we observed that the strength of miRNA suppression of the target protein varies across all the cell lines. Based on percentage of translation repression, cell lines were grouped into three categories viz., (1) 80% or more translation repression which includes cell lines HeLa, COS-7, A549; (2) 50% or more translation repression which includes Hep G2, HUVEC, NIH/3T3, C2C12, U2OS, LNCaP and (3) 20% or more translation repression in HEK-293 cell line. We also assessed the expression of let-7a in transfected cells. Our data clearly show that ectopic expression of wt or mut plasmids did not significantly affect let-7a expression suggesting miRNA specific impact on reporter gene activity (Figure 2E,F). Together, these results show marked inhibition of reporter gene silencing by let-7a.

### 2.3. Let-7a Mediated mRNA Stability Varies across Cell Lines

**Figure 3:**
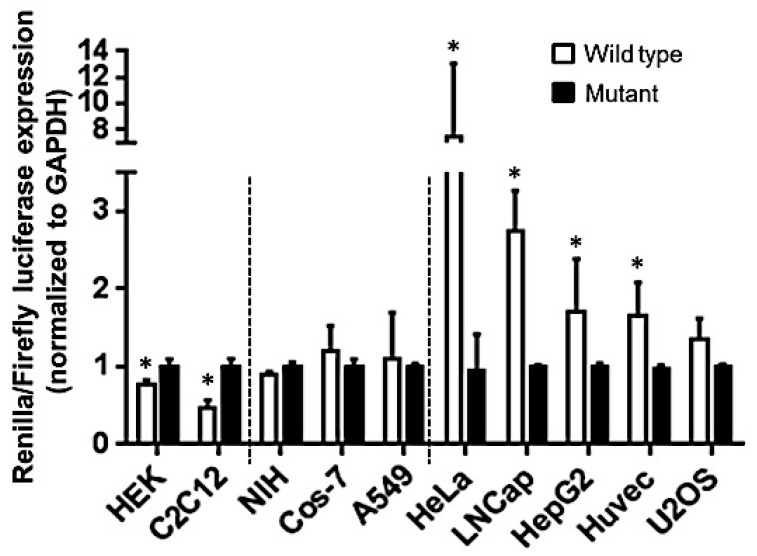
Let-7a mediated degradation of renilla mRNA. Cells were co-transfected with pRLTK-let 7a wt or mut and pGL3 constructs. Thirty-six hours later, total RNA was isolated and reverse transcribed. Real-time RT-PCR indicates the ratio of renilla and firefly mRNA in cells. GAPDH levels were used to normalize the expression level in each experiment. GAPDH normalized ratios of renilla and firefly mRNA is presented as mean ± SD from three independent experiments for each cell line. Dashed lines separate cell lines based on percentage of renilla mRNA accumulation: significantly less (**left**), no change (**middle**), significantly high (**right**). SD value of some samples is too small and therefore not visible on histograms. * *p* value < 0.05, Students *t*-test.

Translation repression is the outcome of both miRNA mediated target degradation or translation inhibition. To dissect whether mRNA degradation or translation repression is the preferred mode of miRNA regulation, we therefore examined the expression of renilla mRNA post-transfection. Unlike protein inhibition, a distinct variation was observed with regard to renilla mRNA accumulation across the cell lines (Figure 3). As seen from Figure 3, we can group cell lines on the basis of their levels of mRNA transcripts. Cell lines which stabilized mRNA transcripts are HeLa, Hep G2, HUVEC, LNCaP, and U2OS. Three cell lines including NIH/3T3, COS-7 and A549 exhibit no significant change in the mRNA transcript, while those that degrade the mRNA transcript were HEK-293 and C2C12. It is well established that miRNA and target gene interaction may stabilize or induce degradation of mRNA [18,19]. Our results indicate that identical miRNA: mRNA interactions are differentially regulated based on cell type.

### 2.4. Differential Accumulation of miRNA-Repressed Messages Is Associated with mi-RISC

**Figure 4:**
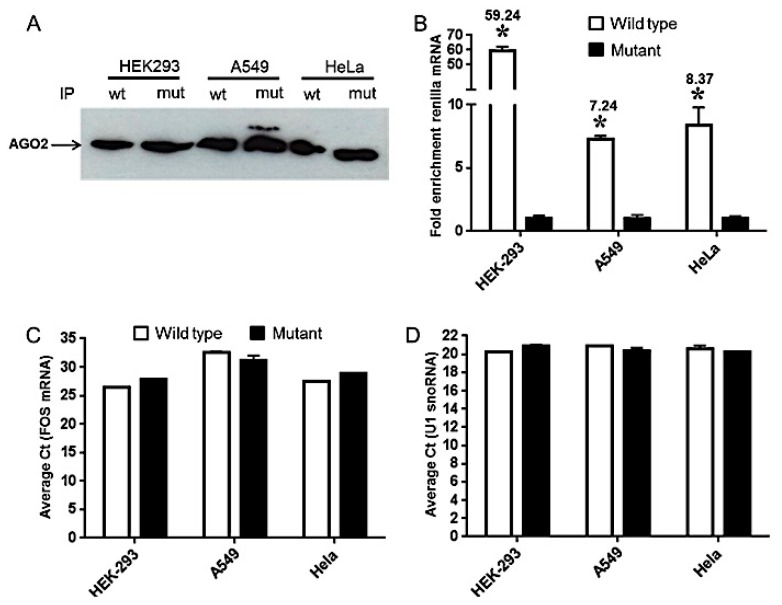
Repressed mRNAs are enriched in Ago-2 immunoprecipitation. HEK293, A549 and HeLa cells were seeded in six-well plates and co-transfected with pRLTK-let 7a wt or mut and pGL3 constructs. Cells were harvested after 36 h and lysates were prepared using cell lysis buffer. Ago-2 IP was performed according to manufacturer’s instructions. (**A**) Western blotting showing detection of Ago-2 in IP of wt and mut plasmid transfected lysates; (**B**) Quantitative RT-PCR of renilla was performed in wt and mut plasmid transfected cells. Results for three independent cell batches are shown. Students *t*-test was performed to calculate significance and * *p* value <0.01 is considered significant; (**C**) qRT-PCR of Ago-2 associated FOS mRNA was used as a positive control for IP. Data represents average *C*_t_ values from three independent experiments; (**D**) As a positive IP control snRNP antibody was used. snRNP bound U1 snoRNA was amplified by qRT-PCR. Data represents average *C*_t_ values from three triplicate experiments. SD values of some samples were too small and therefore not visible on histograms. * *p* value < 0.05, Students *t*-test.

We observed differences in the cellular pools of renilla mRNA, however, this does not reflect the translation inhibition of renilla protein. We therefore assessed the enrichment of miRNA-suppressed transcripts in mi-RISC. To this end, we selected one cell line representing each category (Figure 3) based on the impact on RNA stability as described above (*i.e*., mRNA degradation (HEK293), no change (A549) and mRNA stability (HeLa)). HEK293, A549 and HeLa cells transfected with pRLTK wt or mut constructs were assessed for accumulation of renilla mRNA bound with Ago-2, a crucial component of mi-RISC. Cell lysates prepared from these cells were immunoprecipitated (IP) using human Ago-2 antibody. Western blotting detected Ago-2 and confirmed similar pull-down of Ago-2 in wt and mut plasmid transfections (Figure 4A). We then examined the levels of renilla mRNA in IP samples. Our results show significant enrichment of renilla transcripts in wt compared with mutant mRNA. We found 59.24-, 7.24- and 8.37-fold enrichment of wild type renilla messages in HEK293, A549 and HeLa, respectively (Figure 4B). Ago-2 associated FOS mRNA, a known mi-RISC associated gene, was detected at similar levels in both wt and mut indicating that let-7a mediated accumulation of renilla mRNA into miRISC (Figure 4C). We also used snRNP antibody as a positive control for IP. This protein binds to and pulls down U1 snoRNA. Our RT-PCR results shows highly similar pull down of U1 snoRNA in HEK293, A549 and HeLa (Figure 4D). Taken together, these results further highlight mechanistic differences in miRNA-mediated target silencing.

## 3. Discussion

MiRNA research has rapidly advanced from initial discovery to the stage where miRNA-based procedures are entering the clinic as important diagnostic and promising therapeutic tools. As the central role of miRNAs in disease is being deciphered, their potential as prognostic or diagnostic markers is evidenced by a long list of studies. Indeed, it has been demonstrated that several miRNAs have potential therapeutic value [20,21]. However, several obstacles need to be overcome in order to exploit their value. One key aspect that has largely been understudied is whether a miRNA-target interaction is similarly regulated in different cell types. In the present study, we address this by evaluating let-7a targeted renilla mRNA. Our results show that endogenous let-7a specifically binds to its target gene leading to discrete downstream outcomes depending on the cell type in context. Our finding will have far reaching implications when targeting genes using miRNA as a therapeutic intervention.

All cell lines tested for miRNA regulation of renilla showed significant down-modulation in luciferase activity, implying negative impact on target protein output. However, this could be a consequence of miRNA-mediated translation suppression or target degradation. Our results show reduction in renilla mRNA in HEK293 and C2C12 while, accumulation of target transcript was observed in HeLa, HUVEC and LNCap. In the remaining cell lines, we did not observe significant changes in renilla mRNA levels. Multiple lines of evidences suggest that silencing by a miRNA may either have no impact on the mRNA level or a significantly smaller decrease in mRNA levels than is observed for protein [22,23,24,25]. Further, miRNA targeting with single or multiple binding sites generally trigger translation inhibition or target cleavage, respectively [17,26,27]. The plasmid construct used in this study has two tandem let-7a sites. We noticed that renilla mRNA levels were not altered in NIH-3T3, Cos-7, A549 and U2OS, nonetheless, significant downregulation of protein translation was observed. On the other hand, mRNA degradation was observed in HEK293 and C2C12 while, HeLa, LNCap, HepG2 and Huvec exhibit increased renilla mRNA levels implying transcript stabilization. This observation is in agreement with findings from another study where miR-124 transfected HEK293 cells showed increased mRNA degradation (~35%) of its approximately 600 Ago-2 associated targets along with corresponding protein reduction [28]. Overall, these results indicate that differences in identical miRNA-target interaction may yield cell specific outcome with regard to the mode of miRNA regulation.

Previous studies from our lab highlighted cell specific differences in mRNA and miRNA mediated gene regulation. For instance, primary human Mφ and DC challenged with LPS exhibit transcriptome-wide changes. However, the magnitude of changes in gene expression was extremely high in DC relative to Mφ [15]. Given that miRNAs are global regulators of gene expression; it can be speculated that such differences observed here could be driven by miRNA-mediated gene silencing. This was further supported by our findings that transfection of primary human Mφ and DC with three different miRNAs viz., miR-24, miR-30b and miR-142-3p, clearly show that changes in mRNA levels of genes linked to the phagocytosis pathway were prominently impacted by all three miRNAs in DC and to a much lesser extent in Mφ [16]. Our present study also provides evidence that similar differences exist and are attributed to the underlying mode of miRNA regulation of its target transcripts. For instance, A549 and HeLa showed very strong suppression of renilla activity indicating translation inhibition (Figure 2C). However, renilla wt mRNA was highly stabilized in HeLa (~7.8-fold) but A549 showed no significant differences between wt and mut (Figure 3) whereas Ago-2 IP of renilla mRNA demonstrate comparable accumulation in HeLa and A549 (Figure 4B).

Although this study highlights hitherto unknown complexity in the alternate pathways adopted by miRNA to silence target transcripts, we acknowledge that further studies are required to gain finer details to address these inherent differences. For instance, we examined only one miRNA-target interaction with two binding sites but whether increasing or decreasing the number of target sites will impact the outcome of miRNA function needs to be examined. Similarly, while we evaluated the imperfect miRNA: target interaction, how the strength of interaction between miR and target influence translation inhibition or target mRNA cleavage needs further investigation. It will also be interesting to compare and contrast these variables for various mammalian miRNAs in order to establish consensus mode of regulation.

Two distinct cytoplasmic structures, stress granule (SG) and GW/P bodies have been demonstrated as sites of miRNA bound targets [29,30,31]. Several translation factors including eukaryotic translation initiation factor 3 subunit b (eIF3b), poly(A)-binding protein (PABP), Ras GTPase-activating protein-binding protein (G3BP) are associated with SG indicating these serve as sites for translationally stalled messages [29,30,32]. GW/P bodies primarily co-localize with components of cytoplasmic mRNA degradation machinery including Dcp2 and Dcp1 [30,33]. Moreover, GW bodies are closely associated with Multi-Vesicular Bodies (MVBs). The invagination of MVBs leads to the formation of exosomes. Multiple studies have reported the presence of repressed messages inside secreted exosomes implying packaging of the target bound miRNAs. We examined renilla expression in total RNA and AGO-2 immuno-precipitated RNA and observed noteworthy differences. Higher stability of renilla in HeLa does not correlate with increased accumulation in Ago-2 complexes. Similarly, in HEK293, significantly high mRNA degradation does not corroborate with greater renilla accumulation in Ago-2 IP. These results suggest that increased availability of miRNA bound mRNAs will favor transcript degradation. This is further supported by a report demonstrating that P bodies are formed as a consequence of RNA-mediated gene silencing [34]. Thus, active mi-RISC formation will favor P body formation and hence increased mRNA degradation. Fusion of SG and GW/P bodies has been reported and is mediated by their protein components or cytoskeletal network indicating that mRNA degradation and translation stalling are not strictly spatially distinct processes thereby allowing exchange of miRNA ribonucleoprotein (miRNP) complexes between these cytoplasmic bodies. Taken together, these observations highlight the important and inherent differences among various cell types on miRNA mediated gene silencing. Understanding these complex mechanisms of miRNA target gene silencing can further uncover the role of miRNAs in health and disease.

## 4. Material and Methods

### 4.1. Cell Culture and Differentiation

Ten different cell lines including Hep G2, HeLa, HEK-293, COS-7, NIH/3T3, C2C12, U2OS, LNCaP, A549, and HUVEC were procured from ATCC (Manassas, VA, USA) and cultured according to manufacturer’s recommendations. HeLa, HEK-293, COS-7, NIH/3T3, C2C12, U2OS were maintained at 2 × 10^5^ cells/mL in DMEM, Hep G2 was maintained at 2 × 10^5^ cells/mL in MEM, LNCaP was maintained 2 × 10^5^ cells/mL in RPMI 1640 medium and A549, HUVEC were maintained 2 × 10^5^ cells/mL in F12 K medium. All the 4 media used *i.e.*, DMEM, MEM, RPMI and F12 K were supplemented with 10% FCS and 2 mmol/L l-glutamine, penicillin (100 U/mL) and streptomycin (100 μg/mL).

### 4.2. Plasmid Constructs

Renilla expressing plasmids pRL-TK-let7a (wt) and pRL-TK-let7a (mut) engineered with tandem let-7a binding sites were obtained from Addgene (Cambridge, MA, USA). Firefly expressing pGL3 construct was purchased from Promega (Madison, WI, USA).

### 4.3. Transfection Efficiency of Cell Lines

All the cell lines used in this study were assessed for transfection efficiency using GFP expressing plasmid pGLO (Bio-Rad, Hercules, CA, USA). Each cell line was seeded (3 × 10^4^) in 48-well plates and transfected with 100 ng/well pGLO plasmid using Lipofectamine 2000 (Invitrogen, Grand Island, NY, USA) according to manufacturer’s instructions. After 24 h post-transfection, cells were visualized and images were captured on an EVOS fluorescent microscope (Life Technologies, Grand Island, NY, USA). GFP-positive cells were determined using flow cytometry and the percent positive population was analyzed using FlowJo software (Ashland, OR, USA).

### 4.4. Luciferase Reporter Constructs and Dual Luciferase Reporter Assays

Dual experiments were carried out in a 48-well format. In brief, Hep G2, HeLa, HEK-293, COS-7, NIH/3T3, C2C12, U2OS, LNCaP, A549 and HUVEC cells were seeded at the density of 3 × 10^4^ in respective media supplemented with 10% fetal bovine serum. All transfections were performed in quadruplicate using 0.5 μL Lipofectamine 2000, 100 ng dual luciferase reporter plasmids, 80 ng pGL3. After 36 h post-transfection, cells were lysed in passive lysis buffer (Promega, Madison, WI, USA) and dual luciferase assays were performed using the firefly and renilla substrates provided in dual luciferase reporter kit (Promega, Madison, WI, USA). Readings were obtained on a luciferase plate reader (Biotek, Winooski, VT, USA).

### 4.5. Total RNA Isolation

Total RNA was isolated using the miRNeasy kit (Qiagen, Germantown, MD, USA) following manufacturer’s protocol. The RNA was quantitated using the NanoDrop (Thermo Scientific, Wilmington, DE, USA).

### 4.6. Quantitative Real-Time PCR

For renilla and firefly mRNA quantification, 500 ng total RNA was reverse transcribed using the Superscript RT-II kit (Life Technologies, Waltham, MA, USA). A 20 μL reaction mix was prepared using 2X EvaGreen Master Mix (Biotium, Hayward, CA, USA), ~20 ng of cDNA, and 10 pmoles of each forward and reverse primer. The real-time PCR was carried out in a StepOne 7500 Thermocycler (Applied Biosystems, Carlsbad, CA, USA). GAPDH served as an internal control and all reactions were run in triplicates. The *C*_t_ values of replicates were analyzed to calculate relative fold change using the delta-delta *C*_t_ method. The data are presented as ratio of GAPDH normalized renilla and firefly mRNA expression. For mature miRNA (let-7a) quantification, let-7a miScript primers and miScript II RT Kit were purchased from Qiagen. One hundred nanograms of total RNA was reverse transcribed according to the manufacturer’s instructions. The reactions were run using miRNA specific primers and universal primer in the PCR mix buffer. RNU6 was used as endogenous control.

### 4.7. miRNA: mRNA Immunoprecipitation (RIP)-Chip with Anti-Ago2

For each sample (HEK293, A549 and HeLa), 2 × 10^7^ cell equivalents were subjected to immunoprecipitation using the Magna RIP kit (Millipore, Billerica, MA, USA) following the manufacturer’s protocol. Briefly, approximately 36 h after transfection, cells were washed with ice-cold PBS, scraped off and lysed in 250 μL of complete RIP lysis buffer (provided in the kit). One hundred μL of whole cell extract was incubated with 900 μL RIP buffer containing magnetic beads conjugated with human anti-Ago2 antibody (Millipore, Hertfordshire, UK) or negative control normal mouse IgG (Millipore, Hertfordshire, UK) and rotated for overnight at 4 °C. Samples were washed three times with RIP wash buffer, and 20 μL samples were analyzed by western blotting using anti-Ago2 antibody (1:1000) (Cell Signaling Technologies, Danvers, MA, USA) to check IP efficiency. The remnant samples were incubated with Proteinase K buffer at 55 °C for 30 min with shaking to digest the protein. Co-immunoprecipitated RNA including miRNA:mRNA complexes were subjected to qRT-PCR analysis. Renilla mRNA was assessed in input and IP samples. An Ago2-IP/IgG-IP ratio of >5.0-fold was considered positive enrichment of the Ago2-IP fraction. FOS mRNA amplification (Forward primer: GAGAGC TGGTAGTTAGTAGCATGTTGA; Reverse primer: AATTCCAATAATGAACCC AATAGATTAGTTA) served as positive Ago-2 IP control while, snRNP antibody was used as IP control and associated U1 snoRNA was detected by qPCR.

### 4.8. Western Blotting

Ago-2 was detected in cell lysate or immune-precipitated proteins. Samples were boiled in Laemmli buffer, and the proteins were separated by sodium dodecyl sulphate-polyacrylamide gel electrophoresis (SDS-PAGE). The separated proteins were transferred to a nitrocellulose membrane (Hybond ECL; GE Healthcare Biosciences; Pittsburgh, PA, USA) at constant voltage of 20 V for 20 min. The membrane was blocked with Tris-buffered saline (TBS) containing 5% skimmed milk (Bio-Rad) for 1 h at room temperature and washed with TBST (TBS containing 0.1% Tween 20). The membrane was then incubated overnight at 4 °C with the primary human Ago-2 antibody appropriately diluted in TBST–5% skimmed milk, washed 4 times for 10 min each with TBST, and incubated with horseradish peroxidase-linked secondary antibodies diluted in TBST–5% skimmed milk for 1 h at room temperature. After the membrane was washed as described above, chemiluminescent detection of proteins was carried out using Luminol reagent (Santa Cruz Biotechnology, Dallas, TX, USA) according to the supplier's protocol. Primary anti-Ago-2 and secondary antibodies were supplied as part of IP kit.

### 4.9. Statistics

Data were analyzed using GraphPad Prism (GraphPad Software, La Jolla, CA, USA). Mean values, error bars (standard deviaminutetion), and Students *t*-test (two-tailed) were calculated from three independent experiments. A *p* value of <0.05 was considered significant.
